# The Edifice of Vasculature-On-Chips: A Focused Review on the Key Elements and Assembly of Angiogenesis Models

**DOI:** 10.1021/acsbiomaterials.3c01978

**Published:** 2024-05-07

**Authors:** Joshua Lim, Hsu-Wei Fang, Sasinan Bupphathong, Po-Chan Sung, Chen-En Yeh, Wei Huang, Chih-Hsin Lin

**Affiliations:** † Graduate Institute of Nanomedicine and Medical Engineering, College of Biomedical Engineering, Taipei Medical University, Taipei 11031, Taiwan; ‡ High-value Biomaterials Research and Commercialization Center, National Taipei University of Technology, Taipei 10608, Taiwan; § Department of Chemical Engineering and Biotechnology, National Taipei University of Technology, Taipei 10608, Taiwan; ∥ Institute of Biomedical Engineering and Nanomedicine, National Health Research Institutes, Zhunan 35053, Taiwan; ⊥ School of Biomedical Engineering, College of Biomedical Engineering, Taipei Medical University, Taipei 11031, Taiwan; # Department of Orthodontics, Rutgers School of Dental Medicine, Newark, New Jersey 07103, United States

**Keywords:** angiogenesis, endothelial cells, microfluidic assembly, vasculature-on-chip

## Abstract

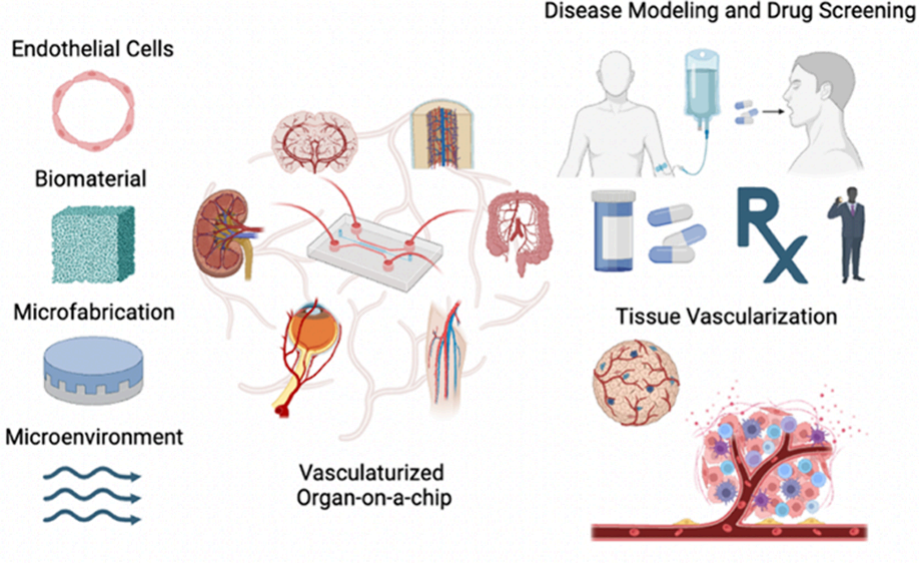

The conception of vascularized organ-on-a-chip models provides researchers with the ability to supply controlled biological and physical cues that simulate the *in vivo* dynamic microphysiological environment of native blood vessels. The intention of this niche research area is to improve our understanding of the role of the vasculature in health or disease progression *in vitro* by allowing researchers to monitor angiogenic responses and cell–cell or cell–matrix interactions in real time. This review offers a comprehensive overview of the essential elements, including cells, biomaterials, microenvironmental factors, microfluidic chip design, and standard validation procedures that currently govern angiogenesis-on-a-chip assemblies. In addition, we emphasize the importance of incorporating a microvasculature component into organ-on-chip devices in critical biomedical research areas, such as tissue engineering, drug discovery, and disease modeling. Ultimately, advances in this area of research could provide innovative solutions and a personalized approach to ongoing medical challenges.

## 1 Introduction

**Figure 1:**
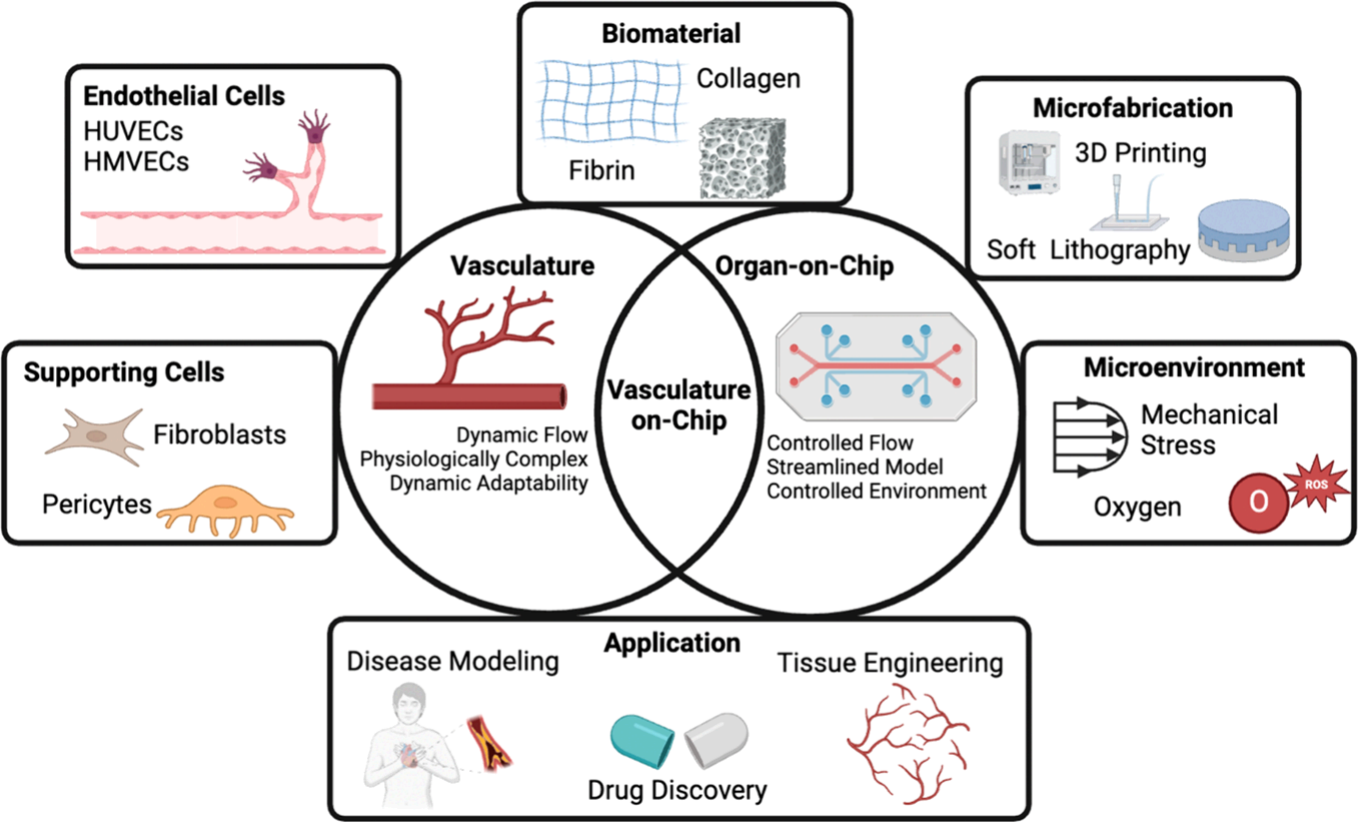
Vasculature-on-chip encompasses a combination of vasculature and organ-on-chip technology. The construction of vascularized organ-on-chip models involves several critical elements, including endothelial cells, supporting cells, biomaterials, device fabrication, and the microenvironment. These components are integral to creating a functional blood vasculature in OoC platforms for disease modeling, drug testing, and tissue engineering. Created with Biorender.com.

The idea behind organ-on-a-chip (OoC) technology is to create a platform that replicates the physiological and functional properties of human organs by combining advances in tissue engineering and microfabrication.^1^ Generally, OoCs are specifically designed to emulate tissue-specific functions by regulating the microenvironments of cells, providing a platform for investigating organ-level physiology, human pathophysiology, and the effect of therapeutics in a controlled *in vitro* setting.^2^ The innovative approach of OoC technology to reproduce physiological functions of various organs, such as the lungs, heart, kidneys, and liver, has emerged as a promising alternative to the traditional animal models.^3−6^ Moreover, they offer several advantages over standard 2D and 3D culture by providing a more physiologically relevant microenvironment for the cells and having the option to facilitate high-throughput experimentation for drug screening applications.^7^ Central to sustaining organ and tissue function is the blood vasculature, an intricate network of blood vessels throughout the body that serves a vital role in maintaining homeostasis by removing metabolic waste products and transporting oxygen and nutrients.^8^ Consequently, significant emphasis was placed on integrating a local microvasculature for OoC systems, which will hereafter be rereferred to as vasculature-on-chip (VoC). VoCs are complex microengineered models designed to mimic the human vascular architecture and dynamic microenvironment of blood vessels in a controlled manner.^9^ These systems enabled the careful examination of several vascular processes such as angiogenesis, vasculogenesis, vessel permeability, and barrier function, which provide valuable insights into vascular biology, disease mechanisms, and drug response.^10−12^ This review provides a comprehensive guide to the critical elements of vasculature-on-chips, including the selection of appropriate cell types, biomaterials, microenvironmental factors, chip design, and standard practices, which are generally considered when assembling organ-on-chip technologies with an integrated microvasculature (Figure 1). The article later explores the applications of such technologies in key biomedical research areas, such as tissue engineering, drug discovery, and disease modeling, with the goal of offering innovative solutions to ongoing medical challenges.

## 2 The Blood Vasculature: Physiological Processes of Vessel Formation

The human blood circulatory system comprises a highly complex closed-loop network of hollow conduits known as blood vessels. They play a crucial role in regulating organ development, maintaining homeostasis, and promoting tissue regeneration by enabling the circulation of blood and diffusion of molecules.^13^ These blood vessels are arranged in a hierarchical branching network and are characterized by diameters ranging from arteries and veins (large vessels:1 cm to 1 mm; small vessels:1 mm to 100 μm), arterioles and venules (20–100 μm), and capillaries (5–20 μm).^14^ Generally, larger vessels are composed of a single layer of vascular endothelial cells (EC), which are encircled by a layer of smooth muscle cells (SMC) and an external layer of fibroblasts and other extracellular matrix (ECM) components.^15^

Arteries and arterioles are primarily responsible for conveying oxygenated blood throughout the body, whereas veins and venules are responsible for eliminating deoxygenated blood and metabolic waste.^16^ The capillary unit, with its distinct morphology comprising a solitary endothelium, is instrumental in facilitating the exchange of metabolites, nutrients, and oxygen between the vascular system and surrounding organs and tissues.^17^ Apart from participating in metabolic functions, the vasculature in diverse organ and tissue microenvironments also assumes a crucial function in maintaining the solute and water balance between blood and tissue sections, reacting to various deformations and stress fluctuations, regulating blood coagulation, and modulating immune trafficking between the bloodstream and peripheral tissues.^18−20^ These functions are primarily carried out by the endothelium or vascular ECs, which are semipermeable single-layer membranes that line the lumen of the vessel walls. Furthermore, the expansion of vascular ECs offers a remarkable ability to sustain life systems by redesigning the blood vessel network to conform to local surroundings and repair vascular injuries.^21^

The fundamental processes involved in the *in vivo* development of blood vessels are primarily attributed to vasculogenesis and angiogenesis. Vasculogenesis refers to the de novo assembly of a primitive blood vascular network from endothelial progenitor cells or angioblasts, which are cells derived from fibroblast growth factor (FGF) induction of mesodermal cells.^22^ Generally, vasculogenesis takes place during the developmental stages within the avascular tissue. However, spontaneous vasculogenic formation can also occur in adults and postnatal children owing to conditions such as ischemia, inflammation, malignancy of mainly multipotent endothelial progenitor cells, and EC recruitment from the bone marrow.^23^ Whereas, angiogenesis refers to the formation of new capillaries from an existing blood vessel through sprouting or intussusception.^24^ The angiogenic process is essential for various biological functions, including cancer growth and metastasis, extracellular matrix remodeling, and embryonic development.^25−27^ Before the onset of angiogenesis, the endothelium has a predetermined pattern for positioning the tip and stalk cells. It is vital to understand that both the tip and stalk cells are not stationary, as they are in constant competition for the position of the tip cell.^28^ Tip cells denote the specialized ECs that lead the migration process by forming lead sprouts and guiding the migration stalk behind them, whereas stalk cells refer to the ECs that follow the tip cells. Typically, ECs, referred to as stalk cells, have a less migratory phenotype but higher proliferation rates. During the onset of angiogenesis, migrating ECs move toward areas where signaling molecules, such as growth factors, are present. These ECs then proliferate and undergo a series of remodeling stages to form hollow channels that make up the new blood vessels.

## 3 Cell Types for Vasculature-on-Chip

### 3.1 Endothelial Cell Types

The primary components of vasculature-on-chips are ECs, which line the luminal surface of the blood and lymphatic vessels. However, the sources of ECs used vary substantially across reports. The human umbilical vein endothelial cell (HUVECs) line is a widely used type of EC isolated from the vein of the umbilical cord. Recently, more reports on HUVECs have detailed the use of commercially available or in-house transfected green fluorescent proteins (GFP) or red fluorescent proteins (RFP).^29,30^ The advantage of utilizing GFP- or RFP-transfected cells is that they allow live cell tracking of the cells inside the chip or encapsulated in the hydrogel without the need to fix and stain the cells. Because GFP and RFP operate at different wavelengths and can be easily differentiated, both types of transfected HUVECs can be used to study EC behavior. Kameda et al. employed this methodological approach in which a human lung fibroblast (HLF)/GFP-HUVEC spheroid was situated atop a formed vascular bed formed by GFP-HUVECs in order to investigate anastomoses and vessel perfusability between the microvascular bed and the tumor spheroid.^30^ Apart from HUVECs, human aortic endothelial cells (HAOECs), which are ECs isolated from the aorta, are another cell line that is widely used for modeling angiogenesis. Seo et al. made a case that under simultaneous microfluidic flow conditions and vascular endothelial growth factor A (VEGF-A) stimulation, HAOECs have significantly better angiogenic potential than HUVECs through the upregulation of fibroblast growth factor-2 (FGF2) and fibroblast growth factor-5 (FGF5), regardless of gel type and matrix stiffness.^31^

Unlike HUVECs, human aortic endothelial cells (HUAECs) are isolated from the arteries of the umbilical cord. Although not as popular as HUVECs, a recent study suggested that there was no significant difference in proliferative activity, cell membrane integrity, and secretion of vasoactive substances between HUAECs and HUVECs in 2D culture.^32^ Another study compared the fluid flow response and mRNA expression levels of CD31, plasminogen activator inhibitor-1 (PAI-1), arterial and venous markers (ephrin-B2 and EphB4), endothelial gap connexin (CX37, CX40, CX43), and fit-related tyrosine kinase (FLK1/KDR and VEGFR) in induced pluripotent stem cell-derived endothelial cells (iPSC-ECs), HUVECs, and HUAECs on a microfluidic platform.^33^ When focusing on HUAECs and HUVECs, researchers found similar PAI-1 secretion and F-actin arrangement between the two cell types; however, higher endothelial gap junction CX protein subunits (CX37, CX40, CX43) and Ephrin-B2 mRNA expression were found in HUAECs, and only higher EpHB4 mRNA expression was found in HUVECs. While a recent study found that activation of the angiotensin-2 receptor induced angiogenesis in HUAECs in a pregnancy-specific manner, to the best of our knowledge, HUAECs are yet to be considered as the endothelial cell type for angiogenesis-on-chip studies.^34^

Primary cells are considered an attractive option for creating vascular models for specific purposes. For instance, Bai and colleagues designed a blood-brain barrier (BBB)-model by isolating ECs and pericytes from the brains of two mouse strains identified as “high” and “low” angiogenic strains to study the interplay between the cell types in a 3D microenvironment.^35^ Using a microfluidic-based platform, Bai was also able to direct soluble factors, specifically VEGF-A, across the hydrogel compartment in which ECs and pericytes were encapsulated to study the processes, properties, and regulation of genes that affect angiogenesis. The location of the source or type of primary EC depends on the platform application. When Yu designed a BBB-model chip, they relied on the use of endothelial cells, pericytes, and astrocytes isolated from rat cerebral cortex pups.^36^ Meanwhile, others have previously opted to utilize EC-differentiated induced pluripotent stem cells (IPSC-ECs) for modeling neurovascular units, the outer blood-retinal barrier, and BBB models.^37−39^

### 3.2 Supporting Cells

While direct inoculation of growth factors and alike on ECs or hydrogels has been proven to induce angiogenesis, the inclusion of supporting cells or additional chambers for it has grown exponentially in recent literature because of their ability to provide vessel stabilization, biochemical cues, and growth factors to endothelial cells.^40,41^ Stromal cells, frequently fibroblasts, are one of the most explored design elements integrated into VoCs.^42,43^ The intention is to recapitulate the proliferative phase of the wound healings3 process and stimulate the secretion of proangiogenic factors such as vascular endothelial growth factors (VEGF), which initiate angiogenic sprouting and regulate vascular formation.^44^ However, it is important to consider the organization of supporting cells within VoCs. Walji et al. investigated the angiogenic sprouting behavior of HUVECs on a microfluidic chip by examining four different organization (2D monolayer, 3D dispersed, young spheroid, and old spheroid) of HLFs.^45^ In brief, they observed that the fibroblast configuration affected angiogenic response. For instance, a local angiogenic response was observed in both young and old spheroid configuration, whereas the 2D monolayer and 3D dispersed configurations demonstrated a more dispersed response. Pericytes, which are stromal cells present around the walls of capillary endothelial cells, have also been widely reported to maintain endothelial network stability and integrity, inhibit vascular permeability, and regulate angiogenic sprouting when cocultured with ECs in angiogenesis chips.^46,47^ This can be attributed to pericytes exhibiting attributes of muscular activity by displaying contractile features similar to smooth muscle cells, which is why they are often referred to as vascular smooth muscle cells.^48^

Other more commonly used cell types to stimulate angiogenesis are tumor cells. The vascular network is a key factor in the development and progression of the local tumor microenvironment (TME), especially in hypoxic environments where tumor cells secrete large quantities of proangiogenic factors to enable the rapid formation of blood vessels to meet the demand.^49^ Depending on the target application, researchers have employed different types of tumor cells, such as adenocarcinoma, glioblastoma, lymphoma, and circulating sarcoma cells, in their OoC models.^50−53^ In recent studies, researchers have found ways to integrate 3D tumor spheroids and organoids to closely resemble solid tumors and simulate the TME on VoC platforms. For instance, Chung et al. devised a 4-channel microfluidic TME platform consisting of a central matrix channel composed of tumor–stroma microspheroids to study the EC-cancer interface by observing angiogenic and lymphangiogenic sprouting.^54^ In response to the microenvironment that was established, it was observed that both blood and lymphatic endothelial cells emerged simultaneously from opposite sides of the fibrin gel and interacted with the tumor spheroid.

Stem cells have also proven to be of significant importance, both through their capacity to differentiate directly into endothelial cells and their ability to release proangiogenic growth factors that facilitate vascular formation.^55^ For example, numerous studies have demonstrated that coculturing adipose-derived stem cells (ADSC) with HUVECs, or the use of ADSCs extracellular vesicle-contained culture medium for HUVECs can promote the cell outgrowth and mature vascular formation.^56,57^ Research has also revealed that ADSCs secrete a significant amount of proangiogenic factors, including tissue inhibitor of metalloproteinase 1 (TIMP1), tissue inhibitor of metalloproteinase 2 (TIMP2), and hepatocyte growth factor (HGF).^56^ Mesenchymal stem cells (MSC) are known to support the process of vascular formation and angiogenesis in ECs. Du et al. demonstrated this by coculturing MSCs from different sources (bone marrow, adipose tissue, perinatal umbilical cord, and placental chorionic villi) with HUVECs.^58^ In brief, their findings revealed that placental chorionic villi-derived MSCs (PMSCs) secreted significantly higher levels of hepatocyte growth factor (HGF) and prostaglandin E2 (PGE2) than other MSC types, while both bone marrow-derived MSCs and PMSCs secreted more VEGF than MSCs from adipose tissue (AMSC) and perinatal umbilical cord (UMSC). Additionally, *an in vitro* tube formation assay in Matrigel revealed that both BMSCs and PMSCs exhibited intact tubular formation compared to AMSCs and UMSCs.

## 4 Biomaterials

Angiogenesis-chip platforms commonly incorporate hydrogels to mimic the 3D interstitial space or ECM of vascularized tissues owing to their biocompatibility, biodegradability, malleability, mechanical versatility, and ability to promote the diffusion of proangiogenic factors.^59^ These hydrogels are then typically aligned in a predesigned compartment, which takes the form of a microchannel, followed by seeding ECs to create a monolayer that mimics the endothelium. Although an array of synthetic and naturally derived hydrogels can support angiogenesis, biomaterials implemented in organ-on-chips remain limited. In Table 1., we summarize a list of hydrogels that have been integrated into vasculature-on-chips.

**Table 1:** List of Biomaterials Utilized in Vasculature-On-Chip Devices^a^

Biomaterial	EC type	Supporting cells	Chip Design	Application	ref.
Collagen	HUVEC	U87 MG cell line	Standard PDMS with Needle templated microchannel	Vessel Co-option Glioblastoma-Vasculature model	(60)
	HUVEC	MSC	Standard PDMS with Needle templated microchannel	3D microvessel model	(61)
	TIME cells	MDA-MB-231	FEP tubing with needle templated microchannel	Vascularized Tumor model	(62)
Fibrin	HUVEC	hiPSC-Neuron, hASC, hBMSC	Three-lane channel	Neurovascular network model	(72)
	HUVEC	NHLF	Open-top design with liquid guiding rail	Vascularized Spheroid platform	(70)
	BMEC	hPC, hAC	Five-lane channel	BBB model	(71)
Matrigel	HUVEC	-	2 reservoirs connected with microchannel	Tumor angiogenesis model	(76)
	HCoMECs	HCT-116	3-phase micro- bioreactor: circular central chamber	Colorectal tumor model	(77)
GelMA	HUVEC	murine 10T1/2 cells	3D printed (PLA) perfusion system with coaxially printed channels	Antiangiogenic drug screening model for tumor application	(82)
DexMA	HUVEC, HMVECs	-	Hydrogel templated parallel microchannel	Platform for studying Cell-matrix interaction for TE material requiring vascularization	(83)

a Abbreviations: U87 MG cell line:; MSC: Mesenchymal stem cell; TIME cells: Telomerase-immortalized microvascular endothelial cells; MDA-MB-231: epithelial, human breast cancer cell line; FEP:; hiPSC-Neuron: differentiated human induced pluripotent stem cell-neuron; hASC: human adipose-derive stem cell; hBMSC: human bone marrow mesenchymal stem cell; NHLF: Normal Human Lung Fibroblasts; hPC: Human Pericytes; hAC: Human Astrocytes; HCoMECs: human colonic microvascular endothelial cells; HCT-116: human colorectal carcinoma cell line; HMVECs: human microvascular endothelial cells.

Collagen is arguably the most widely used hydrogel material for OoC angiogenesis models.^60−62^ One primary factor is that the composition and microstructure of collagen-based hydrogels were found to help regulate vascular network formation.^63^ Other studies have identified that ADSCs can accelerate angiogenesis of ECs through matrix metalloproteinase (MMP)-mediated proteolytic collagen remodeling.^64^ In some studies, modification of the collagen matrices was carried out to improve the results of angiogenic sprouting. This can be achieved through the addition of growth factors, modification of matrix stiffness, or induction of microenvironmental cues.^65−67^

Fibrin plays an important role in the early stages of wound healing as a provisional matrix responsible for halting blood flow.^68^ The inherent bioactive properties of fibrin have been established to facilitate EC migration and accelerate angiogenesis in wounds, wherein cells can rapidly degrade it through the healing process.^69^ Thus, it is an ideal material for tissue vascularization in OoC models.^70−72^ In OoC models, aside from being able to support long-term 3D cell culture, the concentration of fibrin hydrogel has been shown to influence the percentage of vascularized area, branch length, and vessel diameter in the microvascular network.^73^ In other studies, fibrin has been used as a coating material for membranes or sacrificial molds to exploit its bioactive properties.^74,75^

Another prominent material used in angiogenesis-on-chip models is Matrigel.^76,77^ Matrigel is a prominent hydrogel material that contains imperative structural proteins (laminin, collagen, heparan sulfate, etc.) that promote cell differentiation, proliferation, and angiogenesis.^78^ Dai et al. were one of the first to report a recognizable 3-channel (1 gel and 2 media channels) microfluidic device using Matrigel and HUVECs to guide the directional migration of ECs and study the vasculogenic formation and angiogenic sprouting caused by stimulating one side of the media channel with proangiogenic factors.^79^ Meanwhile, other studies have recommended employing Matrigel to create organoid models or as a supporting material to decrease the base material concentration and improve cell invasion.^80^ It is important to note, however, that while the aforementioned materials are widely incorporated in microfluidic systems, other studies have found success in utilizing composite materials^81^ or alternative materials, such as Gelatin Methacrylate (GelMA)^82^ and methacrylated dextran (DexMA).^83^

## 5 Design and Assembly of Angiogenesis Platforms

**Figure 2:**
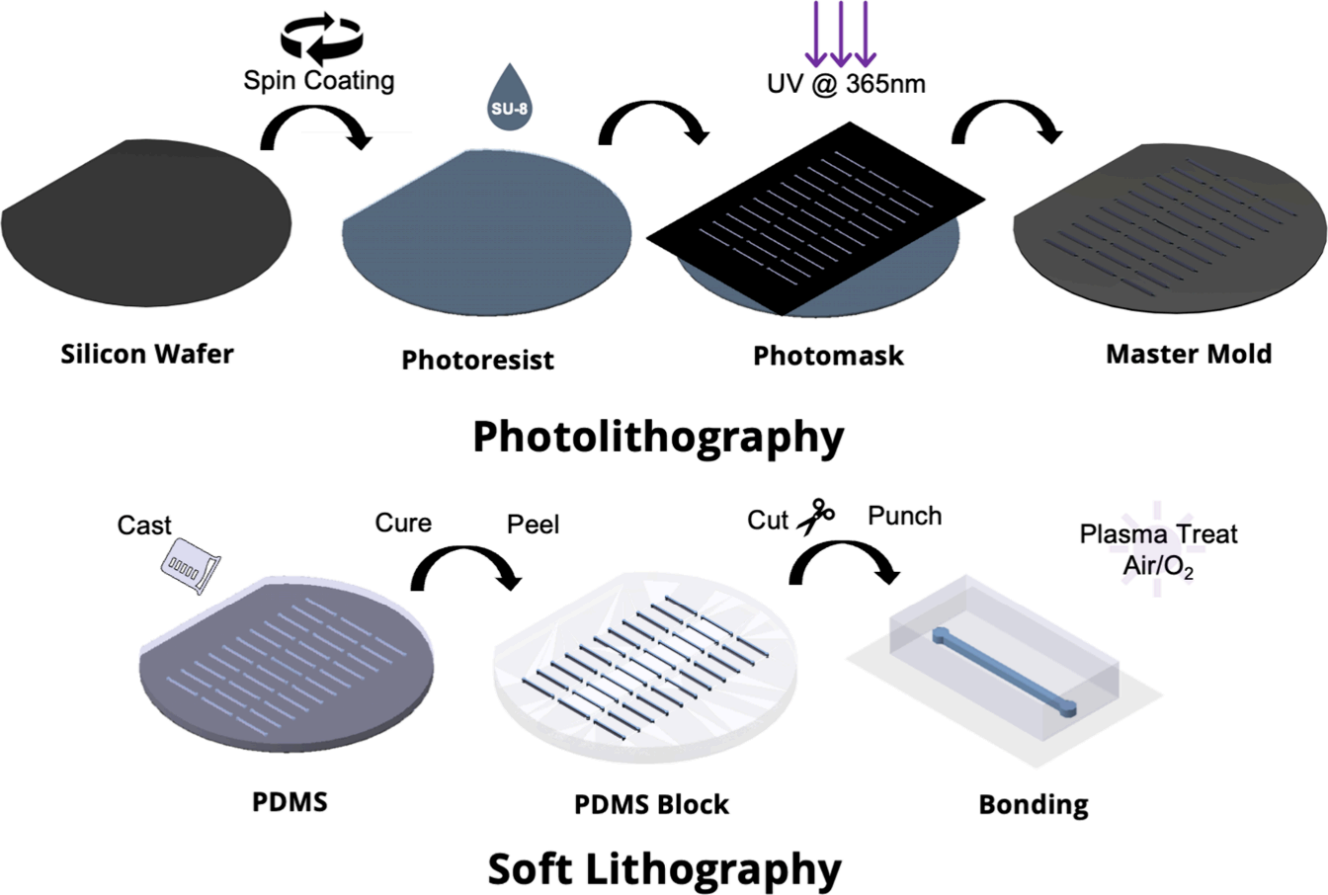
Fabrication of microfluidic devices through combined photolithography for master fabrication and soft lithography for PDMS replication.

Microfabrication has enabled the implementation of innovative methods for manipulating and monitoring cells in artificial environments that closely mimic those found in living organisms.^84^ As shown in Figure 2., this process frequently involves the combination of standard photolithography for generating micro-or nanoscale patterns in master molds and soft lithography, which typically entails the use of polydimethylsiloxane (PDMS), a widely utilized elastomeric polymer for manufacturing microfluidic devices owing to its rapid fabricability, biocompatibility, optical transparency, and gas permeability. Utilizing microfluidic systems also offers the ability to minimize the quantities of reagents and cells used, exercise precise control over spatial and temporal environments, and conduct real-time observation of cellular events.^2^ Moreover, the implementation of compartmentalization has facilitated the scaling up of these systems, making it feasible to screen for more intricate interactions by establishing small and isolated microenvironments while preserving the capacity for crosstalk between chambers.^85^ A prominent practice in VoCs involves multiple channel configurations lined next to each other with pillar arrays, phase guides, membrane barriers, or modular barriers to be the platform for vascular EC lining.^86−89^ In this section, we summarize the employed designs, manufacture, and assembly of current angiogenesis-on-chip models.

### 5.1 Patterned Assembly

**Figure 3:**
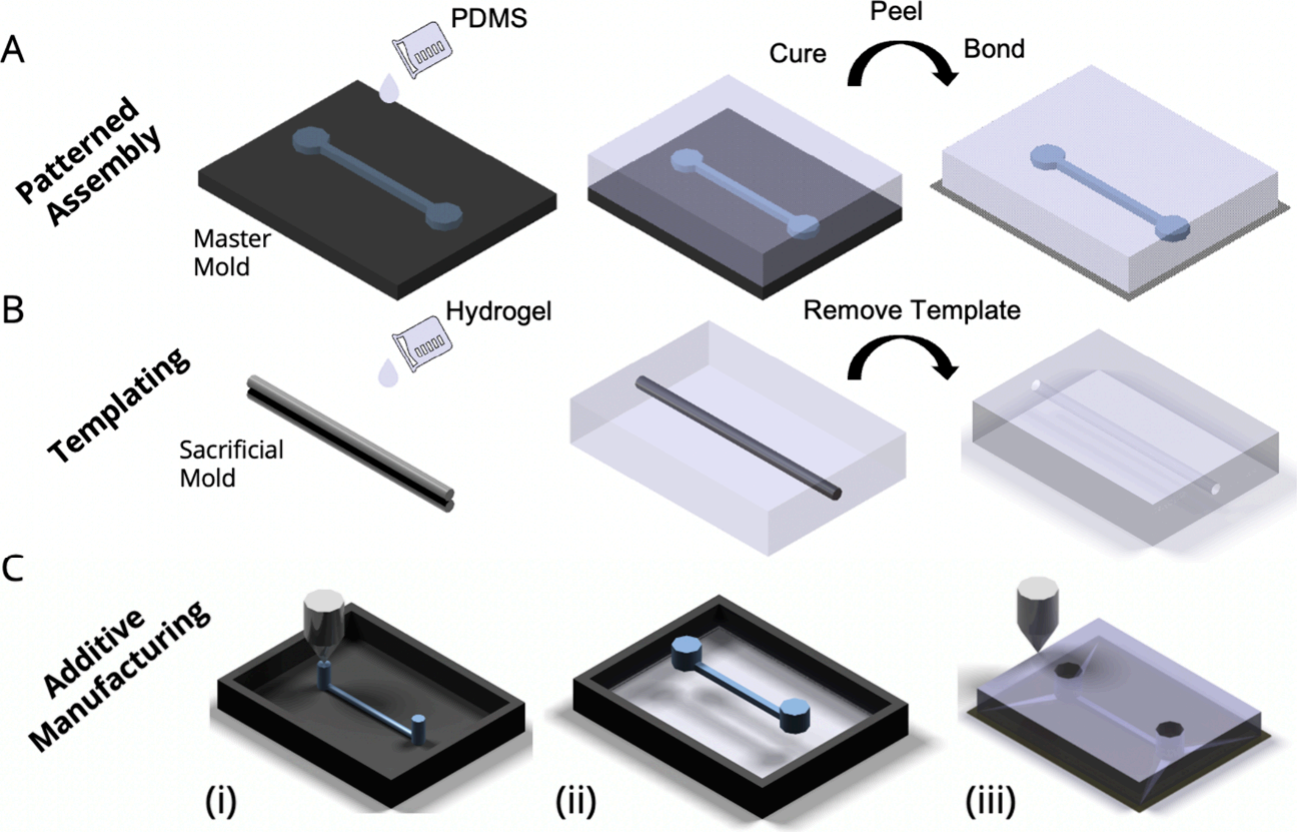
Schematic representation of microfluidic device fabrication. (A) Patterned assembly entails casting PDMS in a master mold. The resulting patterns facilitate the immobilization of hydrogels or membranes, thus providing a surface for EC attachment. (B) Templating involves immobilizing sacrificial molds, such as needles, prior to casting the hydrogel. Upon removal of the mold, a structure resembling the luminal surface of the blood vasculature is generated. (C) Additive manufacturing techniques are frequently employed for three primary purposes: (i) bioprinting alginate as sacrificial templates, (ii) manufacturing 3D printed sacrificial templates or master molds, and (iii) straightaway printing the microfluidic device.

The framework for patterned-assembly chips involves the utilization of a casting-peeling-bonding scheme to create chambers or microchannels to line them with EC-laden hydrogel matrices (Figure 3A). At present, two established techniques are commonly utilized to construct vasculature-on-a-chip devices: (i) vasculogenesis self-assembly and (ii) angiogenic induction in EC monolayers. While the majority of the articles in this review focus on developing an EC monolayer, it is crucial to mention that the employment of EC self-assembly is plausible for developing angiogenesis models. One prominent example is the study by Kim et al., who described the role of interstitial flow in vasculogenic formation and angiogenic sprouting of microvascular networks in 3D cultures.^90^ Their chip design comprises a conjoined center channel, with one side intended for vasculogenesis/microvascular bed formation and the adjacent being the site of angiogenic sprouting, while adjacent to both center channels include media and fibroblast channels to supplement the center channel with growth factors. Analogous iterations of such chip design have also been used to study the effects of growth factors on vessel sprouting, cell–cell interactions, and vessel anastomosis.^86,91,92^ To investigate tumor-induced angiogenesis, open-top chip configurations have quickly gained prominence because of the opportunity to develop better drug screening models by placing tumor spheroids/organoids onto a vascular bed. Oh et al. employed this configuration by devising micropores to separate the cancer spheroid with a vascular bed below it, citing several advantages, such as having the capacity to culture small cell tissues in a microvascular network for extended periods and on a larger scale, and utilizing different fluids to the inner and exterior sides of microvessels.^93^

Modifications to typical pump-driven flow systems have resulted in the development of high-throughput gravity-driven systems capable of screening patient-derived cells.^94^ The implementation of these high-throughput systems has enabled efficient and accurate analysis of large numbers of samples in a short period of time. The use of these systems has significant implications for the advancement of drug discovery and development, enabling researchers to identify and evaluate the efficacy of potential therapeutic agents more effectively and efficiently.^95^ Ko et al. adapted a polystyrene-based 3D spheroid culture platform, termed Sphero-IMPACT, which has a rail-guided center channel that is intended for cell patterning and media reservoir on its sides.^96^ Here, the researchers demonstrated the angiogenic efficacy of the Sphero-IMPACT platform by supplying its center rail with fibrin gel, aligning the HUVECs on the edges of the center rail, and inducing angiogenesis by seeding a U87MG brain glioblastoma cell spheroid onto the center chamber. Commercially available products, such as the OrganoPlate Graft, which is a high-throughput microfluidic system that is attached beneath a standard 384-microtiter plate developed by the company Mimetas, operate in a similar manner. Its design incorporates a central open-top graft chamber intended for grafting organoids or spheroids on top of a hydrogel, and a parallel perfusion channel lined with ECs to mimic the endothelium.^97^ In this system, a distinctive phaseguide design is utilized to generate a boundary-less surface for the ECs to adhere to the hydrogel and sprout toward the center. Similar to previous iterations of the Ogranoplate Graft, its phaseguide technology patterns the hydrogel in the central lane through meniscus pinning, leading to a curved shape contact line.^98^ Ultimately, the intention of high-throughput systems is to accelerate the discovery and development of new therapeutics by enabling massive parallel experimentation.^99^

### 5.2 Templating

Templating with subsequent EC patterning employs a substrate or sacrificial mold to create an endothelium (Figure 3B). A widely utilized approach for creating a luminal endothelial channel is casting a matrix material around a protein-coated small-diameter needle.^75^ After ensuring that the matrix is cross-linked, the sacrificial template will then be carefully extracted and subsequently inoculated with endothelial cells, resulting in an endothelialized luminal surface. Several researchers have utilized this technique by coating acupuncture needles with bovine serum albumin to create a hollow cylindrical structure that represents the blood or lymphatic vessel.^100−102^ Another commonly employed technique for templating is the use of sacrificial agarose molds. Liu and co-workers employed this technique in their lymphangiogenesis model by utilizing bioprinting technology to pattern the microchannel and generate a hollow perfusable conduit within the GelMA constructs which was later embedded with lymphatic endothelial cells.^103^ Templating could also come in the form of pretailoring a scaffold to resemble a luminal structure. For instance, Lai et al. utilized a manufacturing method called 3D stamping to design and develop a well plate-based microfluidic system, termed Integrated Vasculature for Assessing Dynamic Events (InVADE), which features a hollow and suspended vascular interface that provides a means for culturing tissues.^104,105^ By employing a well plate-based microfluidic system, the InVADE platform is able to facilitate the efficient assembly of vascularizing organoids and vascular disease-drug interactions.^106,107^

To create more intricate and complex patterns, researchers have investigated the use of water-soluble sacrificial molds. Goh and Hashimoto successfully showed this by patterning their microchannels by 3D printing poly(vinyl alcohol) (PVA) directly onto several hard and soft substrates/matrices.^108,109^ Despite the inconsistency in the dissolution of sacrificial molds by each substrate, which could be attributed to several variables, such as mold size, area exposed to the aqueous solution, and infill patterns, the integrity and biocompatibility of the infill patterns were preserved. Similarly, Zhang et al. utilized 3D printing technology to create sacrificial templates made from gelatin when they introduced UniPlate, a polystyrene-based microfluidic platform that enables unidirectional media recirculation.^110^ The UniPlate system was paired with a calibrated rocker, which is typically utilized in high-throughput systems to regulate the flow rate, simulating *in vivo* venous perfusion. Templating can also occur in the form of a physical barrier, as described by Bai et al., who used an acrylic barrier that can be assembled in a PDMS device to insert a biocompatible membrane to distinguish between a self-assembled cellular compartment and a growth factor-supplemented acellular compartment.^87^ The motivation for this setup was to create a platform for assessing implantable biomaterial candidates by evaluating EC migration and vascular sprouting across thin biomaterials.

### 5.3 Additive Manufacturing

Creativity through additive manufacturing technology has led researchers to develop a series of angiogenesis models that are extremely difficult to emulate using patterned assembly and templating (Figure 3C). For instance, Salmon et al. developed a custom 3D printed microfluidic system using the Formlabs Dental SG resin, which is aimed at studying the interaction between a human pluripotent stem cell (hPSC)-based cerebral organoid and vascular cells (EC + pericytes).^111^ Here, Salmon illustrated a circular top-sealable construction that can induce angiogenic sprouting from vascular cells encircling the organoid chamber. In contrast, Elomaa et al. developed a 3D printed perfusion system using a similar formlabs resin that enclosed a rolled central perfusable HUVEC cell sheet in a collagen hydrogel.^112^ Although the device was not specifically designed to study angiogenic sprouting, they unintentionally discovered vessels sprouting from the central perfusable HUVEC sheet, suggesting the viability of a cell sheet-based approach for creating angiogenesis models. Another study used AM technology to create a chamber with custom fittings attached to a multiplexed gel-flipping system that generated uniform endothelialization in a perfusable network.^113^ In addition to creating bioreactors, researchers have used AM technology to bioprint scaffolds to mimic the endothelium. Zhang and group, for example, reported such hybrid strategy to create endothelialzed myocardial tissue on a chip by embedding bioprinting hydrogel encapsulated ECs and seeding IPS-derived cardiomyoctes on a custom microfluidic perfusion bioreactor.^114^ Gu et al. developed an antiangiogenic drug screening chip by coaxially bioprinting a cell-laden GelMA/gelatin bioink to form a central endothelialized perfusable vessel.^115^ To create a hollow tubular vessel, the HUVEC-laden GelMA bioink forming the outer layer was cross-linked with blue light, whereas the inner gelatin layer was liquefied to produce a hollow cylinder. The remaining outer GelMA structure was preserved using a custom FDM-printed PCL stent to prevent collapse and jamming. The tube system was then positioned in a custom bioreactor and later cast with hydrogel bulk containing GelMA and VEGF to stimulate angiogenesis.

Additive manufacturing technology has also been adapted to create master molds for PDMS-based microfluidic devices. However, this method has been less adapted because 3D printing materials generally inhibit PDMS curing and impede reliable replication.^116^ Shestra et al. proposed the idea of treating 3D printed molds by following up standard alcohol washing and UV curing to remove residual monomers/oligomers with oxygen plasma treatment and silanization to provide a hydrophobic fluorinated monolayer to prevent PDMS from adhering to the walls.^117^ In 2019, Creative CADworks put forth a chemically modified SLA/DLP resin that is designed to prevent PDMS curing inhibition in 3D printed molds, showcasing the potential for rapid master mold fabrication without the need for laborious standard photolithography or silanization.^118^ Another alternative strategy that employs a more direct approach is to directly 3D print the microfluidic chip.^119^ Homan et al. for instance adopted such approach by directly 3D printing a silicone-based ink to create perfusion gaskets, which were then used to encase their kidney organoids atop an ECM layer.^120^

## 6 Physical Microenvironmental Cues Affecting Angiogenic Sprouting

Stimulating angiogenesis has been viewed as a key strategy for addressing the bottleneck in tissue vascularization.^121^ It is widely known to be regulated by a series of chemotactic stimuli, including growth factors, cytokines, and extracellular matrix (ECM) proteins.^122^ However, several studies have also shown that physical factors play a significant role in vascular processes, such as vascular morphogenesis and angiogenesis.^123,124^ In this section, we focus on the mechanical factors and oxygen microenvironment as drivers for angiogenesis and vascular formation.

### 6.1 Mechanical Factors

Mechanical stimulation and induction of several types of stress in microvascular models have been proven to affect vessel formation, modeling, and spouting. In recent years, there has been an exponentially growing interest in employing microfluidic systems to provide a mechanical force to cells *in vitro*. With the aid of advanced software, such as ANSYS and COMSOL, which can run computational fluid dynamics analysis, the design of microfluidic devices and flow systems can be manipulated to precisely mimic the mechanical forces experienced by blood vessels.^125,126^

#### 6.1.1 Blood and Interstitial Flow

**Figure 4:**
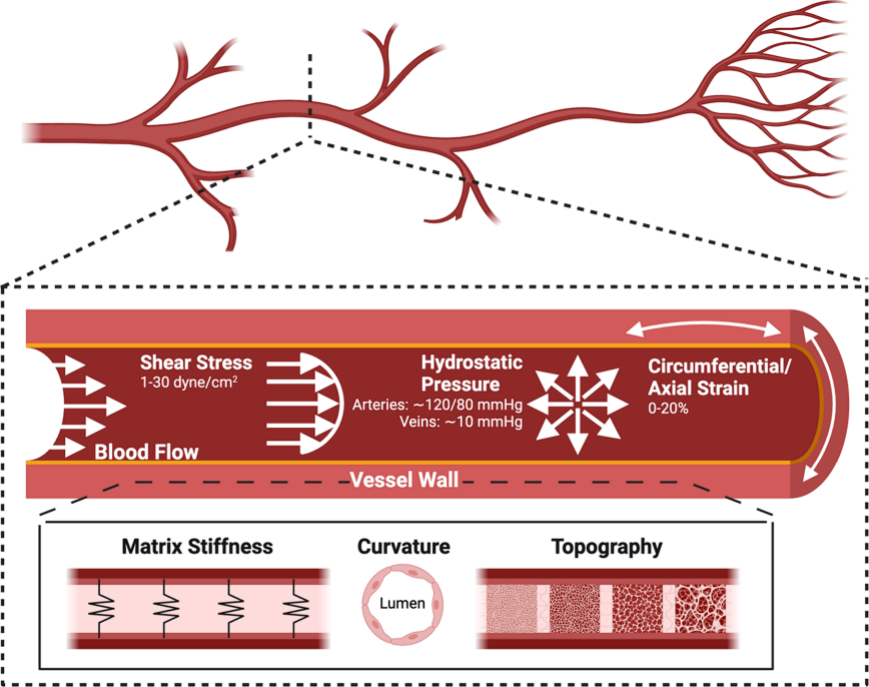
Summary of the biophysical forces that affect blood vessels. Created with Biorender.com.

Despite extensive investigations into the molecular signaling pathways that induce and orchestrate vascularization, biophysical cues have received less attention. Under *in vivo* conditions, mechanical stimulation has consistently been demonstrated to significantly influence neovascularization and angiogenesis.^127^ Blood flow produces mechanical forces that are exerted on vessel walls. It mainly induces three types of mechanical stimuli in the endothelium: fluid shear stress (FSS) along the direction of blood flow, compressive stress caused by the pulsatile nature of blood pressure, and circumferential and axial stretches caused by transmural pressure and tissue movement, respectively (Figure 4).^128^ With the underlying ECM providing additional passive cues, ECs translate perceived mechanical stimuli into biochemical signals to modulate gene expression, protein synthesis, and cell activity (proliferation, migration, and differentiation).^129^ Therefore, VoC systems have been designed to replicate mechanical forces that occur in the cardiovascular system, such as wall shear stress (WSS) in the capillary (10–20 dyn/cm^2^), vein (1–4 dyn/cm^2^), and artery (pulsatile, 4–30 dyn/cm^2^).^130^ Currently, a number of methods are being explored to achieve this. For instance, Kwak and Lee employed a gravity-driven flow in their tumor-VoC system using a platform rocker to generate a laminar shear stress of 3–4 dyn/cm^2^ within the luminal vascular channel.^101^ Meanwhile, Buchanan et al. utilized a pump-driven flow in their tumor-VoC system to investigate the effects of normal (4 dyn/cm^2^), low (1 dyn/cm^2^), and high (10 dyn/cm^2^) microvascular WSS on tumor-endothelial paracrine signaling associated with angiogenesis.^131^ Interestingly, their findings revealed that increasing WSS led to a decreased endothelial permeability.

Applying hydrostatic pressure to ECs *in vitro* has been shown to influence cytoskeletal organization and promote EC proliferation, with previous reports of stimulating angiogenesis and tube formation.^132−134^ Replicating the hydrostatic pressure *in vitro* typically involves simulating the blood pressure, which is the normal force exerted by the blood on vessel walls. However, blood pressure levels differ significantly across the blood vasculature, which may range from approximately 8–10 mmHg in the veins to around 120 mmHg in the aorta during systole.^135,136^ In OoC systems, specifically in pumpless/gravity-driven mechanisms, hydrostatic pressure can be controlled by altering the height of the media reservoir, dimension of microfluidic channels, or by manipulating the incline of the rocking systems.^137,138^ Considering that experiencing hydrostatic pressure is inherent in blood vessels, it is therefore equally essential to maintain the tensile properties of these vessels to preserve their structural integrity and prevent the risk of rupture or leakage. Tensile stresses in the vasculature can be either longitudinal or circumferential. The review article by Camasão and Mantovani examines the hierarchical organization of blood vessels, focusing on their unique structural characteristics, the physiological forces acting on them, and the required mechanical properties when engineering vascular substitutes.^139^ Although no universally accepted standard currently exists for determining the tensile properties of the blood vasculature, numerous *in vitro* studies consider a strain range of 0–20%.^128,140^

Another least-understood biophysical mechanism is interstitial flow. Interstitial flow refers to the mass transport of fluid containing large proteins through the interstitium, blood and lymphatic vessels, and ECM.^141^ It also provides a specific mechanical environment necessary for the physiological activity of interstitial cells.^142^ In angiogenesis chips, researchers have predominantly used such devices to introduce and regulate interstitial flow into their culture system with the aim of better understanding endothelial cell behavior.^90,143^ Deng et al. demonstrated that the coculture of HUVECs and NHLFs under interstitial flow promoted lumenization and perfusability of engineered vasculature, enhanced vascular permeability, and stimulated morphogenesis without the need for additional VEGF.^144^ Interstitial flow has been demonstrated to upregulate matrix metalloproteinase-2 (MMP-2) and promote neovessel formation, demonstrating enhanced vessel formation, connectivity, and function in the brain microvasculature network.^145^ In brain microvasculature models, Winkleman et al. described the role of interstitial pressure by devising a simple 3-channel microfluidic system comprising primary brain ECs, pericytes, and astrocytes within a 3D fibrin matrix.^146^ Alongside observations of the bulk flow of interstitial fluid, which is favorable for angiogenic and vasculogenic conditions, they observed enhanced vessel formation, anastomosis, and longevity in the brain microvasculature model. Although interstitial pressure is driven by the upstream pressure caused by lymph flow, it regulates the interactions between stromal cells and extracellular matrix molecules, ultimately affecting the process of vessel formation.^147^ In addition to interstitial pressure, researchers have widely investigated the use of patterned models to study the effects of cyclic stretching as the primary mechanical stimulus.^109^ Looking into both Ferrari et al. and Zeinali et al., exposure to 3D cyclic mechanical stretching led to differential effects on angiogenesis and de novo vascularization, with higher loads leading to decreased angiogenic sprouting and increased vasculogenesis.^148,149^

#### 6.1.2 Matrix Characteristics

In addition to flow-derived stresses, cell-matrix contact is a fundamental consideration when engineering tissue constructs. Biophysical cues from contact stresses, such as topography, matrix stiffness, and curvature, are well-considered variables that influence EC behavior (Figure 4).^128^ ECs play a critical role in blood vessel function, and the surface roughness of the scaffold can significantly affect the interaction between the scaffold and cells. Scaffold topography plays a crucial role alongside matrix proteins and binding sites in promoting cell adhesion, which is a fundamental requirement for tube formation *in vitro*.^150^ It is well established that cells are sensitive to the topographical arrangement of scaffolds, which ultimately influences the ability of ECs to migrate, proliferate, and differentiate into surrounding tissues.^151^ Thus, optimization of the topographical features of scaffolds to mimic the native microenvironment of the cells involved is necessary to achieve the desired results. Yan et al., for example, examined the influence of different concentrations of GelMA on material morphology and mechanical properties, as it relates to the biocompatibility with rat bone marrow mesenchymal stem cells (rBM-MSCs).^152^ They discovered that a lower concentration of GelMA resulted in increased porosity, optical properties, hydrophilicity, and proliferation and growth of rBM-MSCs. Conversely, a higher concentration of GelMA led to increased mechanical stiffness and a smaller pore size, which in turn led to a significant decrease in the rates of cell proliferation and growth.

The mechanical behavior of the ECM, such as its stiffness characteristics (plasticity and elasticity), is a critical aspect to consider because it has an impact on regulating endothelial cell behavior and gene expression. For instance, Bastounis et al. pointed out that having a stiffer matrix increases EC-matrix traction stress which activates integrin β1, guanine nucleotide exchange factor 2 (pVav2), and 70 kDa ribosomal protein S6 Kinase (p70S6K) for both HUVECs and HMVECs.^153^ Stiffer matrixes also activate p-ERK in HUVECs and activate focal adhesion kinase (FAK) in HMEC-1. In another study, Wei et al. demonstrated that having an optimized gel plasticity facilitates matrix remodeling of ECs.^153,154^ In brief, they found that medium plasticity produces the largest tubular lumen, longest EC invading distance, and highest sprout number when compared to low and high gel plasticity. Previously, we also demonstrated that altering ECM stiffness modulates cell adhesion, migration, proliferation, and differentiation, which are crucial in angiogenesis.^155^ Aside from endothelial cells, the stiffness of the extracellular matrix (ECM) may also influence the behavior of other cells, which in turn impacts EC behavior. Bao et al. demonstrated that ECM stiffness directly influences neuroblastoma angiogenesis by regulating the expression and secretion of VEGF_165_ via the YAP-RUNX2-SRSF1 signaling axis.^156^

A close inspection of the cross-section of blood vessels would reveal that ECs situated in the endothelium conform to the contour of the luminal wall. Although the effect of curvature on EC junctions remains relatively underexplored, the contribution of shear stress to the formation and maintenance of these structures cannot be overlooked. In fact, Kim and colleagues have demonstrated that the role of shear stress relative to curvature is critical in determining cell function in the context of tumor cells.^157^ For endothelial cells, Mandrycky et al. demonstrated that a downward 3D spiral curvature template, in which HUVECs were seeded on the luminal surface, caused unique phenotyping and transcriptional changes that may have been generated by the distinct wall shear stress and stress gradient of the model.^158^ The template was created using a standard off-the-shelf stainless-steel spring as a sacrificial mold to generate a luminal surface with a spiral geometry. Moreover, Huang et al. emphasized the advantages of using a helical microchannel template over straight channels in terms of cell viability and perfusability.^159^

### 6.2 Oxygen Microenvironment

In addition to mechanical stress, regulation of the oxygen microenvironment plays a role in vascular activity.^160^ Reactive oxygen species, which are a small group of reactive molecules, play a critical role in various cellular functions and biological processes.^161^ In vascular physiology, ROS are essential for maintaining homeostasis, normal vascular function, and smooth muscle contraction.^162^ However, excessive production of ROS can result in damage to vascular cells owing to elevated oxidative stress and can lead to various chronic conditions, such as hypertension.^163^ To mediate this, researchers have opted to integrate compounds with antioxidant properties, such as glutathione, into their system to mediate the oxidative stress experienced by cells. Generally, there are two approaches to address this, which is applying antioxidant pretreatment either in hydrogels or in cell culture.^164,165^ However, with OoC technology, researchers have had the autonomy to precisely control the oxygen microenvironment in cell culture systems.^166^ Hsu et al. demonstrated this by developing an intricate microfluidic device capable of producing oxygen gradients to systematically study its effects on the angiogenic sprouting of ECs.^167^ The results indicated that the sprouting length of ECs significantly increased under low oxygen tension, indicating the critical role of EC sprouting in 3D matrices. Another key research area involving manipulation of the oxygen microenvironment can be traced to angiogenic chips under hypoxic conditions, which have been found to improve endothelial cell barrier function and increase vascular formation.^168,169^

## 7 Standard Validation Tests for Vasculature-On-Chip Systems

### 7.1 Angiogenic Sprouting and Vessel Formation

**Figure 5:**
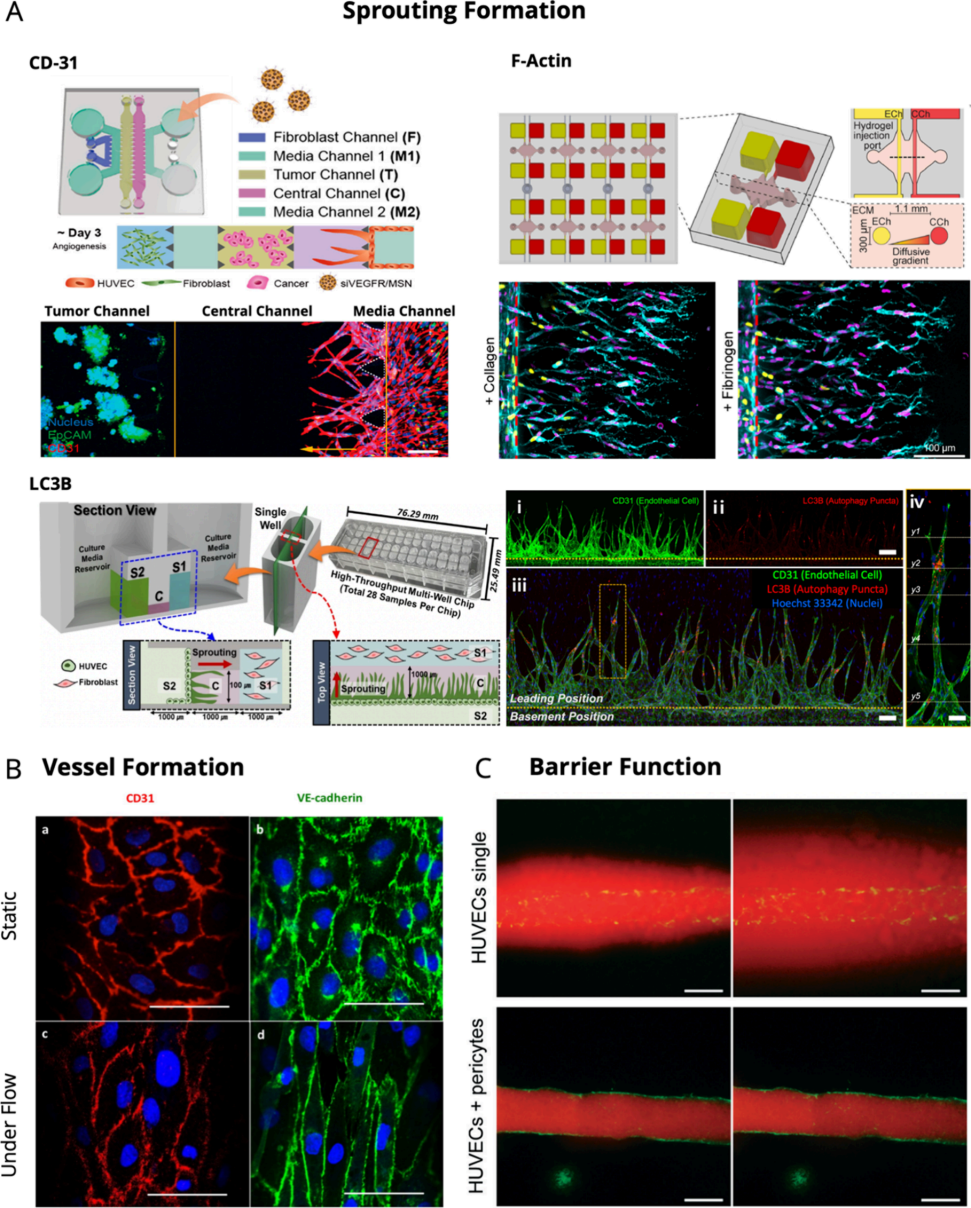
Angiogenic Sprouting and Barrier Formation assays. (A) Sprouting Formation. CD31 (Red: CD31, Green: EpCAM, Blue: Nucleus): A 6-lane microchannel system featuring a parallel outer fibroblast channel, an inner media channel, and a central channel composed of HepG2 cancer cells and space for endothelial cell sprouting. Reproduced from with permission from ref (172). Copyright 2021 American Chemical Society. F-actin (Cyan: F-actin, Yellow: VE-cadherin, Magenta: CD31): Multiplexed angiogenesis-chip showcasing a parallel design with an endothelialized parent channel (ECh) and chemokine channel (CCh) to generate a pressure gradient. Reproduced with permission from ref (173). Copyright 2021 Elsevier. LC3B (Red: LC3B, Green: CD31, Blue: Nucleus): High-throughput gravity-driven angiogenesis-chip which features two parallel media reservoirs and a central channel where hydrogel is patterned. Reproduced with CC BY 4.0 license from ref (174). Copyright 2024 Springer Nature. (B) Vessel Formation: EC morphology under static and flow conditions. Reproduced with CC BY 4.0 *license from ref.* 181. Copyright 2018 Springer Nature. (C) Barrier Function: Dextran-rhodamine diffusion assay conducted on the vessel lumens of a HUVECs and HUVEC/pericyte coculture at 1 min (left) and 30 min (right) post infusion. Reproduced with CC BY 4.0 license from ref (182). Copyright 2020 The Royal Society of Chemistry.

Angiogenic sprouting involves the migration of ECs toward areas where new blood vessels are required, and their proliferation leads to the formation of sprouts that eventually remodel themselves and prune nonanastomosed vessels to create functional and efficient networks.^170^ Investigating angiogenic sprouting and vessel formation is the primary criterion for evaluating the functionality of fabricated angiogenic chips. Angiogenic sprouting assays typically entail the stimulation of endothelial cells to form new vessel-like structures that are capable of mimicking the physiological processes of angiogenesis. Across the majority of literature, researchers have utilized immunofluorescence staining techniques to map EC migration, neo-angiogenesis, and vessel formation patterns. Cluster of differentiation 31 (CD31), also known as platelet endothelial cell adhesion molecule-1 (PECAM-1), is a hemophilic cell adhesion molecule that is highly expressed on the surface of endothelial cells and is involved in several physiological processes in the vasculature.^171^ This protein expression has also been used to quantitatively measure sprout length, number of branches, and vessel density.^172^ In addition to CD31, sprout formation can be assessed by various other means, including the detection of autophagy markers such as LC3B, which may be observed near the tips of sprouts, or by directly examining cytoskeletal components such as F-actin (Figure 5A).^173,174^ Other studies have utilized genetically encoded fluorescent proteins, such as mCherry or Venus-GFP, to label the EC and easily map out EC activity.^175^ However, utilizing transfected or transduced fluorescent protein cells may cause overexpression of fluorescent signals and may hinder the visibility of sprouting formation, as experienced by Elomaa et al.^112^ Nonetheless, fluorescent protein-transfected ECs remain a valuable tool, especially in 3D culture, given that researchers are no longer obliged to shoulder the expense of utilizing expensive antibodies.

Assessing vessel formation likewise involves monitoring the maturation of sprouts into functional vessels.^97^ Interendothelial junctions are protein complexes found between the surfaces of endothelial cells, which play an important role in mechanotransduction and homeostasis.^176^ Adherens junction (AJ) and tight junction (TJ) proteins are the two major types of junction proteins described in endothelial biology. AJ proteins are primarily responsible for mediating cell–cell adhesion via the actions of nectins and cadherins.^177^ Typically, VE-Cadherin (or Cadherin-5) and Nectin-2 are the most common AJ proteins labeled in 3D culture and angiogenesis chips, as they also play a key role in vascular endothelial growth factor receptor (VEGFR) functions and various other cellular processes.^178^ Meanwhile, the main task of TJ proteins is to regulate the passage of small molecules and ions to between ECs and establish cell polarity.^179^ In this end, the zonula occludens-1 (ZO-1) is generally used to label tight junction proteins as they have also been identified to regulate EC migration, barrier function, actomyosin organization, and angiogenesis.^180^ As shown in Figure 5B, Sfriso et al. utilized CD31, VE-Cadherin, and F-actin to illustrate the result of applying shear stress from a pulsatile flow in the arrangement of porcine ECs.^181^

### 7.2 Barrier Formation: Lumen Formation, Vessel Permeability, and Anastomoses

As the EC sprouts toward the surrounding tissue, it slowly takes the shape of a hollow tubular structure, which eventually forms a lumen. Determining the formation of hollow luminal structures of ECs in angiogenesis chips is used to evaluate whether the device can mimic the physiological mechanisms of blood vessels, such as mass transport. While most research groups evaluate lumen formation based on fluorochrome-conjugated antibodies and IF staining, this is typically followed by utilizing fluorescence tracers, such as fluorescein isothiocyanates (FITC), rhodamine, Texas red, and quantum dots, to traverse and illuminate the path of the formed EC lumen in 3D culture.^125,182^ In conjunction with GFP-HUVECs, van Dijk et al. employed rhodamine B-labeled dextran to evaluate the endothelial barrier function of their model, which comprised an ECM channel seeded with HUVECs and neovessels composed of HUVECs and pericytes (Figure 5C).^182^ Their findings indicate that channels that do not provide coverage with HUVEC and HUVEC/pericytes are more susceptible to dextran leakage into the extracellular matrix microenvironment, even after a minute of dextran injection. However, coverage of ECM with HUVECs only likewise produced considerable leakage a minute and 30 s when compared to HUVEC/pericytes.

Multichannel microfluidic devices have also been shown to be capable of manipulating pressure gradients between the media chambers in order to diffuse particles perpendicularly toward the gel channels. For instance, Yue et al. varied the hydrostatic pressure in parallel medium chambers to diffuse FITC-dextran particles through the tissue chamber to validate the perfusivity, lumenization, and anastomosis of the formed microvascular network.^183^ Perfusing fluorescent beads, as described by Liu et al., could also be utilized to quantify lumenized sprouts and visualize their permissible perfusion distances.^83^ Assessment of the vascular bed through particle tracing has also been a useful assessment tool for evaluating vessel-induced damage *in vitro*. Bonanini et al. demonstrated this using the OrganoPlate graft platform by inducing angiogenic sprouting on HUVECs attached to the ECM gel by the induction of a proangiogenic growth factor cocktail.^97^ Upon grafting the hepatocyte spheroids and organoids into the graft chamber, the resulting angiogenic sprouting from each side was anastomosed, resulting in a stable and perfusable vascular network. Subsequently, the vascular bed was exposed to azathioprine (AZA) to simulate veno-occlusive diseases, and the results from the diffusion of FITC-dextran markers demonstrated congestion of the microvasculature due to AZA exposure.

### 7.3 Vasoactivity Markers

At the onset of angiogenesis, cells secrete specific signaling molecules and express key gene markers associated with mRNA, which provides researchers with indicators to predict the initiation of angiogenic sprouting. One of the major indicators of latent angiogenic sprouting is the presence of growth factors, primarily the VEGF protein family, which has garnered significant attention owing to its pivotal role in both physiological and pathological angiogenesis.^184^ The VEGF family consists of glycoproteins segmented into VEGF-A, VEGF-B, VEGF-C, and VEGF-D. Among these, VEGF-A, which consists of several isoforms named after the amino acid they contain, is widely recognized as the central mediator that regulates angiogenesis and vascular permeability.^185,186^ These VEGF-A isoforms are characterized by alternative splicing events to generate homodimeric isoforms, with each having its own distinct properties and function.^187^ For instance, VEGF_121_ and VEGF_165,_ which both signal through VEGFR-2 receptors, are known to be VEGF isoforms with the most abundance and are identified to regulate vessel diameter and promote sprouting formation.^188^ Thus, the detection and quantification of this specific growth factor has been routinely implemented and correlated with angiogenic sprouting. In another study, Liu et al. carried out a comparative analysis aimed at investigating the influence of various VEGF isoforms, in conjunction with interstitial flow stimulated by a microfluidic chip, on promoting angiogenic sprouting and vessel formation.^86^ Here, it was demonstrated that VEGF_165_ displayed a greater ability to promote angiogenesis and vessel formation when compared to VEGF_121_ and VEGF_189_. Some studies have also pointed out that, while VEGF-B plays a similar yet less pronounced function as VEGF-A, its role remains unclear and has been a widely debated area of interest.^189−191^ Meanwhile, VEGF-C and VEGF-D which are the other two primary members of the VEGF family are primarily known for their role in lymphangiogenesis.^192,193^

Another growth factor of interest is FGF, specifically FGF-2, which is a potent mitogen for ECs that induces angiogenesis.^194^ As the major source of FGF, the primary role of fibroblasts is to modulate ECM proteins to produce and remodel the ECM network. Hence, they have been regarded to influence angiogenesis and lumen formation.^195^ On the other hand, platelet-derived growth factors (PDGF) exert a potent influence over cells of mesenchymal origin (e.g., fibroblasts and SMC) and the developing nervous system (e.g., pericytes) serving as both a mitogen to stimulate cell growth, chemoattractant to draw cells toward a particular location, and a survival factor to help cells survive.^196^ Moreover, PDGF exist in two isoforms that initiate signaling of two tyrosine kinase receptors, PDGFRs -β and -β, that stimulates angiogenesis by upregulating production of VEGF and regulating the proliferation and recruitment of perivascular cells.^197^ While both growth factors play significant roles in angiogenesis, this complex physiological phenomenon also involves vital contributors like angiopoietins 1 and 2 which plays a key role in vessel formation and stabilization, TGF-β which regulates EC proliferation and ECM deposition, TNF-α which stimulates the production of angiogenic factors from other cells, and MMP-2 and MMP-9, which are critical in vascular remodeling and sprout formation.^45,198^

## 8 Application of Vasculature-On-Chip Platforms

### 8.1 Disease Modeling and Drug Screening Tools

**Figure 6:**
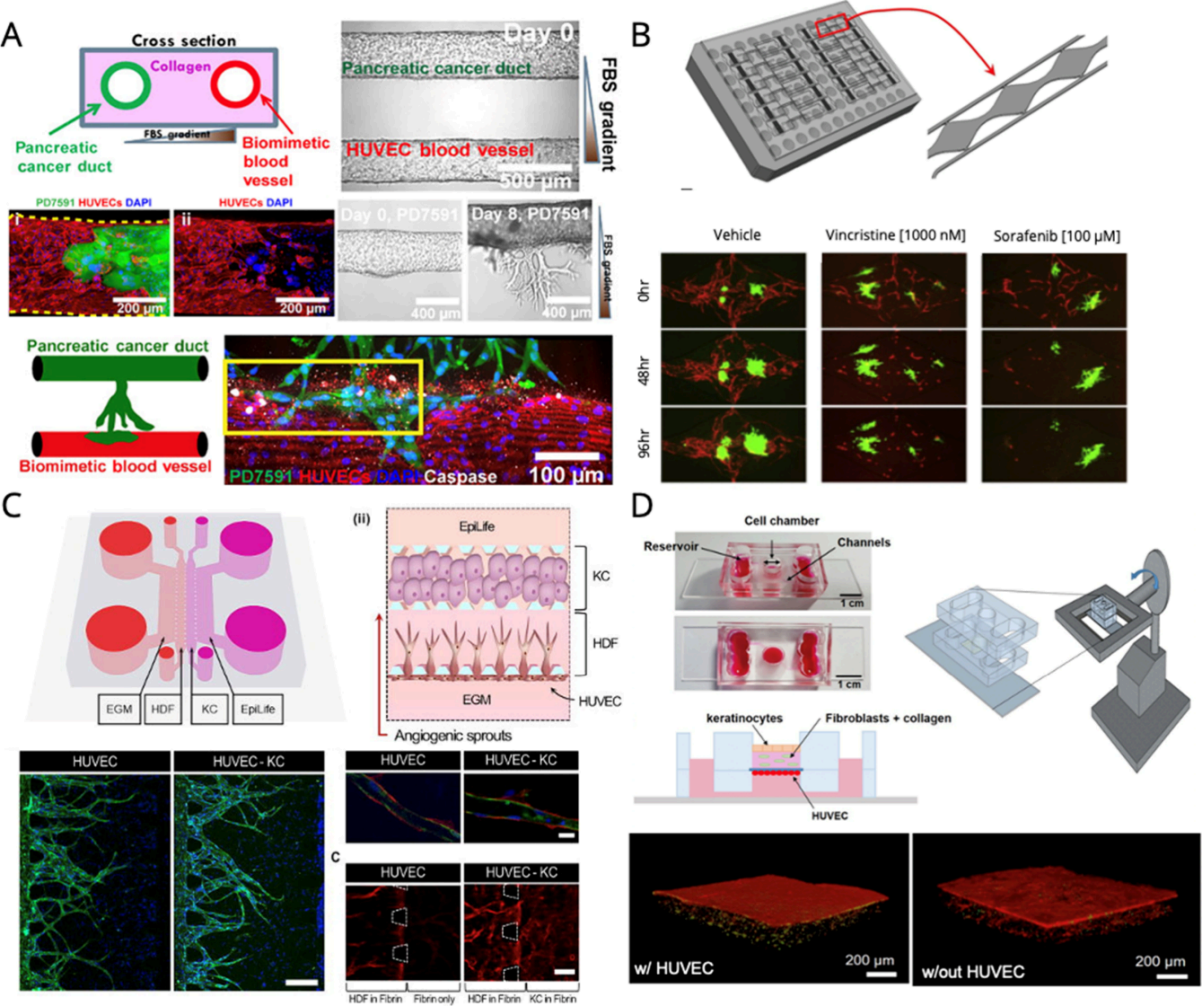
Application of vasculature on chip technology for drug screening and disease modeling application. (A) Organotypic PDAC-on-chip model designed to mimic tumor vascular invasion. Reproduced with CC BY 4.0 license from ref (201). Copyright 2019 American Association for the Advancement of Science. (B) Human vascular microphysiological systems (96-well plate design with PDMS microfluidics and transparent membrane bonded to a bottomless plate) to study anticancer drug effects on HCT116 and EPC-AD cells. Reproduced from with permission from ref (202). Copyright 2020 Elsevier (C) Microfluidic skin-irritation model that leveraged the angiogenic response to assess skin-irritation (HDF: Human Dermal Fibroblasts, KC: Keratinocytes). Reproduced with CC BY 4.0 license from ref (204). Copyright 2019 American Institute of Physics. (D) Schematic of gravity-flow based skin chip, while also showing toxicity of doxorubicin with and without HUVECs. Reproduced from with permission from ref (205). Copyright 2020 John Wiley and Sons.

The primary objective of research focusing on the development of OoC models is to reduce our dependence on animal models for evaluating the effectiveness of novel drugs and therapeutics by providing alternative platforms that will allow researchers to monitor the efficacy of these interventions. In particular, VoCs can be employed to replicate the microenvironment of diseased vascularized tissues and determine potential therapeutic interventions while forecasting therapeutic efficacy. One of the most prominent uses of angiogenesis chip research is to recreate the tumor microenvironment, understand the role of tumor angiogenesis in cancer progression, and measure drug-induced toxicity and angiogenic inhibition.^199^ For instance, Lee et al. developed a three-dimensional microfluidic platform that demonstrated the induction of tumor angiogenesis by various cancer cell types, as well as its subsequent inhibition using mesoporous silica nanoparticles as carriers for small interfering RNA (siRNA) targeting either vascular endothelial growth factor (siVEGF) or its receptor (siVEGFR), which function as RNA-interference-based antiangiogenic nanomedicine (Figure 5A).^172^ While Lee et al. demonstrated a synchronized outcome between their developed *in vitro* and *in vivo* platforms regarding the antiangiogenic and tumor inhibitory effects of siVEGFR/MSN, other studies have proposed the idea of directly harvesting tumor cells from their origin. For example, Wan et al. developed a dynamic vascular tumor-on-a-chip platform using an approach that entailed affixing excised tumors generated in mice onto a collagen matrix to simulate the transport of chemotherapeutic drugs in the tumor vasculature and evaluate the efficacy of individual or combination therapies.^200^ The extracted tumors were facilitated by grafting fragments of human breast tumors onto mice to promote tumor proliferation. Alternatively, Nguyen et al. used an organotypic pancreatic ductal adenocarcinoma (PDAC)-on-a-chip model seeded with PD7591 primary mouse pancreatic cells and HUVECs individually on parallel cylindrical channels to investigate the mechanisms of tumor hypovascularity and the role of the ALK7 pathway (Figure 6A).^201^ In brief, the researchers identified that the activin-ALK-7 pathway mediates endothelial ablation by PDAC. High-throughput microfluidic systems are viewed as highly valuable tools that can contribute significantly to the advancement of new therapies and treatments owing to their ability to expedite the screening and testing of potential medications, enhance experimental control, and minimize costs. Liu et al. demonstrated this by creating a highly reproducible 96-well plate-compatible platform containing human vascularized micro-organs and microtumors, which can be employed to assess the efficacy of various anticancer drugs such as Fluorouracil, Vincristine, and Sorafenib (Figure 6B).^202^ Moreover, this study not only highlights the anticancer effects in the tumor model *in vitro* but also showcases their impact on the microvasculature. Ultimately, realizing success in such endeavors could potentially lead to personalized patient treatment by accurately predicting clinical outcomes and treatment responses *in vitro*.

While cancer research remains to be the primary driving force behind the development of angiogenesis-on-chip platforms, other fields of study are also exploring the potential applications of this technology. The cosmetic industry, in particular, has witnessed a significant rise in the development and application of microfluidic devices, which has been driven by the need to replace traditional animal models with more advanced biomimetic alternatives, such as skin chips.^203^ Jusoh et al. presented a novel *in vitro* skin-irritation model that utilizes the response of angiogenesis in a microfluidic platform as an alternative method for analyzing irritation (Figure 6C).^204^ The model was designed to promote autocrine and paracrine communication by incorporating dermal fibroblasts, keratinocytes, and HUVECs, while irritation was induced using sodium lauryl sulfate (SLS, a common chemical irritant) and steartrimonium chloride (SC, an uncommon irritant). In brief, exposure of keratinocytes to SLS and SC revealed a notable increase in angiogenic formation, which closely resembles the eczematous reaction that frequently occurs in irritant contact dermatitis. Mohamadali et al. developed a microbioreactor human skin chip model, which comprised a normal skin tissue with both the full-thickness dermis and epidermis layers, that mimic the architecture of the microvascular networks as nutrient transporters to the skin layers.^94^ The developed microbioreactor model was found to maintain the characteristics of normal skin and allows for the observation of retinoic acid the diffusion through the tissue, as opposed to the standard tissue culture plates typically used in such studies. Meanwhile, Kwak et al. likewise developed a human skin model composed of dermal fibroblasts and keratinocytes with a vascular endothelium to examine the immune response of human skin tissue (Figure 6D).^205^ Given the crucial role of the vascular endothelium in leukocyte migration toward sites of inflammation, the researchers provoked a basic immune response utilizing sodium dodecyl sulfate and UV-irradiation in their model and assessed the anti-inflammatory effects of dexamethasone. With leukocytes present in the circulating media to simulate the migration pattern of neutrophils, the researchers discovered that the system exhibited an increased secretion of cytokines and revealed a noticeable migration of neutrophils in response to external stimuli, suggesting the potential of the device to mimic the immune response of human tissue.

### 8.2 Tissue Engineering and Vascularization

The development of stable vascular networks is a crucial initial step in the creation of organ-on-chip systems that are designed to have a vascular component, as it is essential to ensure consistency and reliability during drug testing. While there is no single strategy for this, studies have most likely employed the use of proangiogenic factors to induce angiogenesis. VEGF is a widely reported potent regulator of angiogenesis that promotes EC growth, guides migration, and maintains cell survival.^206,207^ Its role has also been demonstrated in pathological OoC platforms such as in tumor and wound healing models to convey the association between the upregulation of VEGF and angiogenesis.^204,208^ While many studies have previously incorporated supporting cells into OoC systems to release proangiogenic factors, some studies have reported promoting angiogenesis by concocting a growth medium composed of VEGF and other proangiogenic molecules such as sphingosine 1-phosphate (S1P) and phorbol 12-myristate 13-acetate (PMA) to stimulate angiogenesis of HUVECs where they observed steady and anastomosed microvascular sprout formation.^209^ Meanwhile, Nashimoto et al. proposes that a sequential addition of fibroblasts to tumor spheroids may enhance vascularization and more accurately model the highly vascularized tumor microenvironment present *in vivo*.^210^ Lee and colleagues who developed a high-throughput microfluidic platform, referred to as U-IMPACT, implemented a similar coculture model to emulate the tumor microenvironment for angiogenesis, vasculogenesis, and tumor cell migration by having a designated spheroid zone sandwiched between parallel tissue channels.^211^ Beyond its high-content and high-throughput screening capabilities, the U-IMPACT platform possessed a unique characteristic wherein it employs a novel patterning method leveraging capillary action to create a tissue microchannel devoid of physical barriers. The issue of vascularization remains a significant challenge in the field of tissue engineering, particularly in the context of engineering entire organs.^212^ The successful integration and survival of engineered tissues or organs within the body is dependent on ensuring proper vascularization. By mimicking the *in vivo* dynamic microenvironment of the vasculature, VoC platforms can provide a controlled environment in which researchers can gain a better understanding of the mechanisms and stimulating factors that encourage vascularization.

## 9 Future Directives and Conclusion

VoC platforms provide a cost-effective and versatile means for investigating vascular processes, such as modeling angiogenesis-related diseases and screening for compounds that either promote or inhibit these processes. The prospect of replicating and scaling up such systems is noteworthy because they could expedite translational research findings from the bench to the bedside, ultimately enhancing patient outcomes and improving existing clinical practices. With numerous vascular-related diseases stemming from microvascular dysfunction, a key challenge in manufacturing VoCs lies in developing innovative design strategies and biofabrication techniques capable of generating reliable microscale *in vitro* models that will accurately mimic cellular interactions and physiological responses. The present review addresses the intricacies of simulating angiogenesis in VoC systems by emphasizing the role of key elements, such as endothelial cells, supporting cells, biomaterials, biofabrication strategies, and microenvironmental factors. This review also attempts to consolidate common practices in validating angiogenesis-based VoC models and highlight their diverse range of applications in various fields, including disease modeling, drug screening, and tissue engineering.

While significant progress has been made in the development of VOC models, minimizing our reliance on animal models would require addressing massive challenges, such as improving device fidelity and forming multiorgan systems with an intricate vascular network. Additionally, standardizing protocols for validating certain microfluidic systems and establishing regulatory frameworks are necessary to ensure the reproducibility and reliability of VOC models. Achieving these objectives could potentially reform how we study vascular biology and supersede current therapeutic practices by advocating for personalized diagnostic and therapeutic interventions.
